# Plant Adaptation and Soil Shear Strength: Unraveling the Drought Legacy in *Amorpha fruticosa*

**DOI:** 10.3390/plants14020179

**Published:** 2025-01-10

**Authors:** Hao Jiang, Xiaoqing Chen, Gang Xu, Jiangang Chen, Dongri Song, Ming Lv, Hanqing Guo, Jingyi Chen

**Affiliations:** 1 State Key Laboratory of Mountain Hazards and Engineering Resilience, Institute of Mountain Hazards and Environment, Chinese Academy of Sciences, Chengdu 610299, China; chenjg@imde.ac.cn (J.C.); drsong@imde.ac.cn (D.S.); 2 Key Laboratory of Mountain Hazards and Earth Surface Processes, Institute of Mountain Hazards and Environment, Chinese Academy of Sciences, Chengdu 610299, China; lm@imde.ac.cn (M.L.); guohanqing20@mails.ucas.ac.cn (H.G.); chenjy@imde.ac.cn (J.C.); 3 School of Life Science and Engineering, Southwest University of Science and Technology, Mianyang 621010, China; xugang@swust.edu.cn

**Keywords:** adaptive strategy, drought-rewetting process, root plasticity, root reinforcement, soil shear strength

## Abstract

Climate change has led to an increasing frequency of droughts, potentially undermining soil stability. In such a changing environment, the shallow reinforcement effect of plant roots often fails to meet expectations. This study aims to explore whether this is associated with the alteration of plant traits as a response to environmental change. Focusing on *Amorpha fruticosa*, a species known for its robust root system that plays a crucial role in soil consolidation and slope stabilization, thereby reducing soil and water erosion, we simulated a drought-rewetting event to assess the legacy effects of drought on the soil shear strength and the mechanical and hydrological traits associated with the reinforcement provided by *A. fruticosa*. The results show that the legacy effect of drought significantly diminishes the soil shear strength. Pretreated with drought, plant roots undergo morphological alterations such as deeper growth, yet the underground root biomass and diameter decline, thereby influencing mechanical reinforcement. Chemical composition analysis indicates that the plant’s adaptation to drought modifies the intrinsic properties of the roots, with varying impacts on different root types and overall reinforcement. Concurrently, the stomatal conductance and transpiration rate of leaves decrease, weakening the capacity to augment soil matric suction through transpiration and potentially reducing hydrological reinforcement. Although rewetting treatments aid in recovery, drought legacy effects persist and impact plant functional attributes. This study emphasizes that, beyond soil matric suction, plant adaptive mechanisms in response to environmental changes may also contribute significantly to reduced soil shear strength. Consequently, ecological restoration strategies should consider plant trait adaptations to drought, enhancing root systems for soil conservation and climate resilience.

## 1. Introduction

Global climate change has disrupted rainfall patterns, causing significant spatiotemporal variations. This has led to frequent short-duration heavy rainfall events, which trigger geological disasters such as landslides and debris flows [1,2,3]. This issue is particularly pronounced in regions experiencing severe soil erosion and ecological vulnerability due to drought, posing a significant threat to human lives, property, and critical infrastructure. As a strategic response, afforestation is expected to enhance soil stability through water retention, reduction in surface runoff, and soil reinforcement via root anchoring [4,5,6,7].

However, the occurrence of large-scale shallow landslides in well-vegetated areas has sparked a debate on the actual capacity of vegetation to reinforce soils. Shallow landslides typically have a sliding surface near the slope surface, usually at a depth of 1–2 m, while plant roots span a wide range of depths, with the majority concentrated around 1 m [8]. The mechanical reinforcement is provided by shallow roots. It functions in a similar way to adding physical supports such as steel or fibers in traditional engineering. By doing so, it significantly enhances the load-bearing capacity of the soil structure. On the other hand, hydrological reinforcement occurs as plants grow. It concentrates on managing water flow and moisture to reduce erosion and improve stability. Together, they ensure structural integrity in diverse conditions [9,10,11,12,13].

The role of different root systems in soil reinforcement is nuanced. Fine roots offer greater tensile strength due to their chemical composition [14], while large roots, particularly those of trees, contribute more significantly to slope stability. Small roots, however, have minimal influence on slopes at the tree or stand scale [15].

Although many studies have highlighted the contribution of plant roots to soil stability, the changes in critical plant traits related to the shear strength of vegetated soil are often overlooked. Using root traits such as biomass, architecture, and chemical composition to predict soil strength captures the system′s instantaneous characteristics, typically long before soil destabilization occurs. This approach treats the dynamic root system as a static anchor, neglecting the phenotypic plasticity of roots under changing conditions [16,17]. Recent studies have begun to recognize the effects of root growth and decay on the shearing behavior of vegetated soils, emphasizing the crucial role these factors play in determining soil shear strength [18,19,20].

In the stabilization of shallow soil, the contributions of mechanical reinforcement and hydrological enhancement by plant roots are pivotal. However, soil moisture conditions significantly impact landslide stability [21,22], particularly through the cycles of drought and rewetting [23]. Previous studies indicated that fluctuations in soil moisture affect matric suction in unsaturated soils, a key factor in maintaining soil stability [24,25,26,27]. From the perspective of unsaturated soil mechanics, negative pore water pressure or matric suction increases due to soil moisture loss through evaporation or transpiration during drought. Conversely, when soil is rewetted, matric suction decreases as soil saturation increases, potentially leading to slope instability. Vegetation can remove soil water through evapotranspiration, increasing soil′s cohesive forces against mechanical failure [11,28,29]. However, the plastic responses and changes in plants during drought-rewetting events and their effects on soil shear strength remain unclear.

*Amorpha fruticosa*, characterized by its robust root system, is highly effective in soil consolidation and slope stabilization, thereby mitigating soil and water erosion. In addition, the plant’s dense foliage contributes to the amelioration of the microclimate and offers a habitat for diverse organisms. Consequently, it plays a significant role in both soil and water conservation and the restoration of ecological balance. In this study, *A. fruticosa* plants were subjected to a simulated drought-rewetting event to evaluate the legacy effects of drought on soil shear strength provided by plants and the morphological and chemical traits associated with mechanical and hydrological reinforcement. We hypothesize that plant adaptive strategies during the drought-rewetting process affect the performance of traits related to mechanical and hydrological reinforcement, thereby weakening the plants’ ability to maintain soil shear strength. This investigation into the legacy effects of drought on soil shear strength and plant traits could provide crucial insights into the complex interactions between plants and soil under changing environmental conditions.

## 2. Materials and Methods

### 2.1. Experimental Materials and Designs

The experiment was carried out at the Yanting Agro-ecological Experimental Station of Purple Soil, the Chinese Academy of Sciences (Yanting County, Sichuan Province, China, 31°16′ N, 105°27′ E). The climate of the area is of mid-subtropical monsoon climatic type with annual average temperature and precipitation being 17.3 °C and 826 mm, respectively. The soil for growing plants is typical purple soil developed from Jurassic purplish shale, which is widely distributed along the Yangtze River.

**Figure 1:**
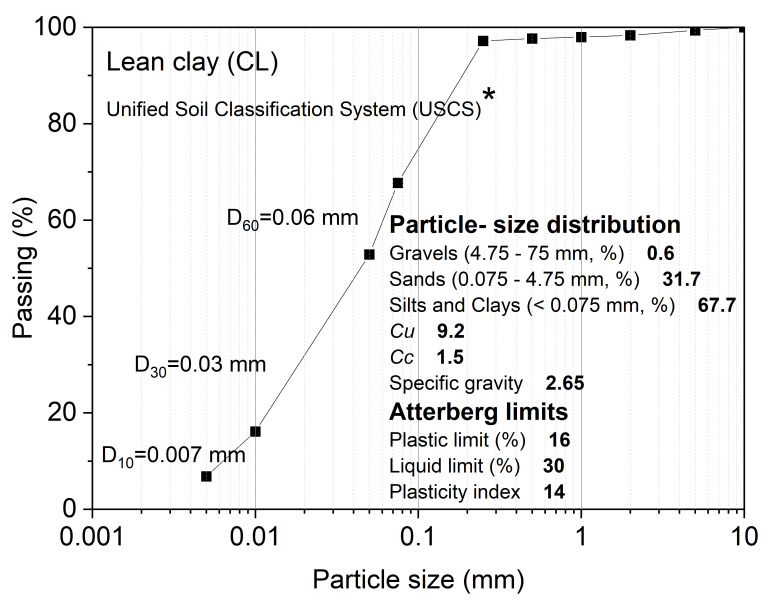
Cumulative curve of particle size distribution. * According to the Unified Soil Classification System (USCS, ASTM D2487-17), the soil used in the present study was classified as lean clay (CL). *Cu*, coefficient of uniformity (*D*_60_/*D*_10_); *Cc*, coefficient of curvature.

Before planting, soil was manually homogenized, and large stones and roots were removed. The dry density of 1.28 g cm^−3^ represents the normal dry density of this particular soil type in the natural environment of the study site. This density could provide better water permeability and aeration properties, thereby promoting plant growth and root development. The particle size distribution curve is plotted in Figure 1. According to Atterberg limits and the Unified Soil Classification System (USCS; ASTM D2487-17 [30]), the soil is classified as lean clay (CL). The chemical properties of the soil are as follows (based on kg^−1^ dry soil): pH is 8.2; organic carbon is 6.06 g; total nitrogen is 0.68 g; and available phosphorus is 2.67 mg.

In April 2021, a total of eighty one-year-old plants of *Amorpha fruticosa* were transplanted into polyvinyl chloride (PVC) vegetative test cylinders with an inner diameter of 390 mm, one plant per pot. The test cylinders were designed in a bifurcated manner, consisting of an upper chamber with a depth of 260 mm and a lower chamber of 200 mm depth. To ensure optimal water drainage, the bottom plate of each cylinder was perforated with several 5 mm diameter holes, a modification validated in previous studies [31]. After a two-month acclimation period, 20 plants with uniform stem diameter (approximately 5.8 mm on average) and height (approximately 42 cm on average) were carefully selected. The experimental design included three distinct treatments. Firstly, a drought treatment was applied to ten plants. This treatment entailed withholding irrigation for a period of 60 days. As a result, the plants showed clear symptoms of being stressed by drought. Subsequently, a controlled rewetting phase was implemented. These plants are hereinafter referred to as the ‘previous drought’ group. Secondly, the remaining plants were subjected to a treatment that simulated average rainfall conditions. Specifically, 70 mm of water was allocated per month. This group is hereinafter referred to as the ‘non-drought’ group. Thirdly, to serve as a control for soil conditions without plant influence, five additional pots without plants were established and designated as the ‘bare soil’ group. We added water to those pots to match the soil volumetric water content of the ‘non-drought’ group. To better restore the natural state of the soil, the soil was settled after filling and not compacted. For monitoring volumetric water content, EC-5 soil moisture sensors (Decagon Devices, Inc, USA) were placed 200 mm from the base of the cylinders. These sensors were integral to the precise measurement of soil moisture throughout the experimental period. Prior to the initiation of the soil direct shear test (October 2021), all samples were irrigated until the soil reached the saturated water content level of 100%, and then the water supply was stopped. When the volumetric water content of the soil decreased to approximately 35% (during this period, the air humidity was relatively high and the plant transpiration was weak, so this volumetric water content was relatively stable in a short time), the plants were harvested for morphological, physiological, and chemical analysis.

### 2.2. Direct Shear Tests

A series of undrained direct shear tests were conducted according to Ghestem et al. [4] and Chen et al. [32] with minor modifications. Before testing, aerial parts of the plants were removed. Accounting for soil compaction due to plant growth and irrigation, soil height in the vegetated test cylinders was measured. The vegetated test cylinder was carefully placed inside the direct shear box using an electrical winch. In this study, the direct shear box contains two parts: upper and lower boxes. Both were 200 mm deep and there was a 10 mm gap between them to ensure horizontal separation of the two parts during shearing. In this study, the initial normal stress is about 3 kPa, which comes from the soil weight of the upper box. No additional vertical load is applied because it is considered that in the actual landslide, the normal stress on the sliding zone is only the self-weight stress of its overlying soil layer.

Based on a series of preliminary experiments, it was observed that when the volumetric water content was 25%, the soil direct shear test exhibited favorable results. After shearing, the soil in the upper part of the direct shear box can still maintain a relatively complete shape, and most of the collected soil parameters are normal. Therefore, this volumetric water content of 25% was established as the reference standard for the soil direct shear test. However, due to weather conditions, specifically higher air humidity, and the different plant performance in each vegetated test cylinder (resulting in differences in transpiration), it is challenging to attain the same volumetric water content (approximately 25%) for each treatment within a short timeframe. Consequently, only two replicates of each treatment were conducted to reflect the approximate differences in soil tangential shear stress between treatments. In the present study, shear tests were carried out at a speed of 0.075 mm s^−1^ and the total lateral displacement was fixed at 80 mm. The decreasing shear plane area during the test was taken into account when calculating the stress–strain curves.

(1) $$\tau =\frac{T}{S}$$

(2) $$S=\frac{1}{2}(d^{2}*arccos\frac{\gamma }{d}-\gamma \sqrt{d^{2}-\gamma^{2}})$$

*τ* is the tangential shear stress (kPa); *T* is the measured tangential force (kN); *d* is the inner diameter of the vegetated test cylinders (*d* = 390 mm); and *γ* is the tangential displacement (mm). It should be noted that we are cognizant of the pore water pressure variation during undrained shear, and pore water pressure is generated in the shear zone. In this study, our objective is to demonstrate the changes that occur in soil shear strength, with a focus on analyzing the changes in plant traits associated with shear strength and speculating on their potential relationship to changes in shear strength.

### 2.3. Prediction of Soil Shear Strength by the Wu and Waldron Model (WWM)

The WWM [33,34] was used to predict the shear stress of soils in this study. Specifically, the shear strength of the soil (*τ_s_*) is as follows:

*τ_s_*_=_*c* + σ tan *φ* + ΔS*_r_* (3)

The cohesion (*c* = 11.24 kPa) and internal friction angle (*φ* = 37.2°) were determined by the Mohr–Coulomb criterion [32]; σ is the effective vertical stress (σ = 3 kPa); ΔS*_r_* is the increased shear strength (kPa) due to roots and can be computed as:

ΔS*_r_* = *t_r_* (sin *θ* + cos *θ* tan *φ*) (4)

where *t_r_* (MPa) is the average mobilized tensile strength of roots per unit area of the soil and *θ* is the angle of root distortion in the shear zone. In general, the values of (sin *θ* + cos *θ* tan *φ*) can be approximated as 1.2 for 30° < φ < 40° [33]. Thus, Equation (4) can be simplified as:

ΔS*_r_* = 1.2 *t_r_* (5)

and *t_r_* can be calculated as:

(6) $$t_{r}=\sum_{i=1}^{n}T_{ri}(\frac{A_{ri}}{A})$$

where *A_ri_/A* is the root area ratio (RAR, dimensionless). *A_ri_* is the cross-sectional area (in mm^2^) of each root, whereas *A* is soil area (in mm^2^). *T_ri_* (MPa) and *d_ri_* (mm) are the tensile strength and diameter of an individual root (*i*), respectively. *T_ri_* can be calculated according to an empirical tensile strength–diameter correlation equation derived from the same species reported by Bai et al. [35]:

(7) $$T_{\mathrm{ri}}=48.55\;d_{ri}^{-0.82}$$

The shear strength values obtained from the direct shear tests were compared to the predictions using the WWM prediction at 60 mm tangential displacement.

### 2.4. Plant Morphology

After harvesting, plant samples were divided into roots, stems and leaves. Biomass samples were dried (80 °C, 48 h) to constant weight and weighed. The root/shoot dry mass ratio was calculated. Specifically, the entire root system of each plant was carefully removed from the PVC vegetated test cylinder by flushing them with tap water. In this study, the developmental approach was used to make a root-order-based classification [36]. Briefly, the most proximal roots originating from the embryo, hypocotyl/mesocotyl or shoot (i.e., tap, basal or shoot-borne root) are typically considered as zero-order roots, while the distal roots in the system would then be the highest-order roots. Parameters related to root architecture were obtained using the WinRHIZO software (WinRHIZO Pro 2007, Regent Instruments, Inc., Québec, Canada). Root weight ratio (RWR, g g^−1^), specific root length (SRL, cm g^−1^) and root tissue density (RTD, g cm^−3^) were calculated.

### 2.5. Gas Exchange

Gas exchange was measured using the fully expanded and intact young leaves near the shoot apex of each plant. Net CO_2_ assimilation rate (*A*), transpiration rate (*E*), and stomatal conductance (*g*_s_) were measured with Li-Cor 6400, a portable photosynthesis measuring system (Li-Cor Inc., Lincoln, NE, USA). The gas exchange was measured in the morning (08:00–11:00). The following conditions were used: leaf temperature, 25 °C; leaf-to-air vapor pressure deficit, 1.5 ± 0.5 kPa; photosynthetic photon flux (PPF), 1000 μmol m^−2^s^−1^; relative air humidity, 65%; and ambient CO_2_ concentration, 350 ± 5 μmol mol^−1^. The photosynthetic water use efficiency (WUE) is the ratio of carbon gain in photosynthesis to water loss in transpiration, i.e., *A*/*E*.

### 2.6. Root Chemistry

The samples of roots were ground and passed through a 20-mesh screen after being dried (60 °C, 48 h) to constant weight by repeated measurements. The root carbon concentration (RCC, mg g^−1^) and nitrogen concentrations (RNC, mg g^−1^) were determined by the semi-micro Kjeldahl method as described by Kost and Boerner [37], respectively.

Nonstructural carbohydrate (NSC) concentration was defined as the sum of soluble sugar and starch concentrations, which were determined as described by O’Brien et al. [38]. Briefly, root samples were ground, and 15 mg of each sample was used for NSC analysis. Soluble sugars were extracted with a shaking bath of 80% ethanol at 27 °C overnight, followed by two additional 2 h extractions [39,40]. The remaining starch was digested with amyloglucosidase. The concentrations of soluble sugars and starch (measured as glucose equivalents) were determined by spectrophotometry at 487 nm after a phenol–sulfuric acid reaction.

The cellulose concentration in the roots was determined as described by Leavitt and Danzer [41]. Root samples were ground into a fine powder, weighed, and inserted into a Soxhlet apparatus fitted with a flask containing a mixture of 99% toluene and 96% ethanol (2:1; *v*/*v*) with a volume of 700 mL. The mixture was heated to boiling point. After 24 h of extraction, the samples were removed from the Soxhlet and placed in distilled water heated to 100 °C for 6 h. Next, a solution of 700 mL pure water, 7.0 g sodium chlorite (NaClO_2_) and 1.0 mL acetic acid (C_2_H_4_O_2_) was poured into a beaker and mixed with a magnetic stirrer. The solution was heated to 70 °C for 18 h. Then, the mixture was removed from the Soxhlet apparatus and submerged in boiled distilled water for 6 h. The mixture was rinsed with distilled water, oven-dried at 60 °C for 24 h, and weighed. The percentage of cellulose was evaluated by computing the weight difference between the initial and final weights of each sample, relative to its initial weight.

Root lignin concentration was measured using the ADL (acid detergent lignin) method [36,42,43]. First, dry glass tubes of approximately 20 mL were weighed and about 250 mg of a milled sample of roots was added. Then, 10 mL of a solution consisting of 2% cetyl trimethylammonium bromide in 1 L of 0.5 M H_2_SO_4_ was added, and the tubes vortexed and refluxed for 1 h at 100 °C. The tubes were vortexed regularly during the reflux. The residue was centrifuged at 2500× *g* for 10 min and the supernatant was removed. The residue was then washed four times with hot water (15 mL) and twice with acetone (15 mL). The residual acetone was evaporated by placing the glass tubes in a water bath at 60 °C overnight. The tubes were then dried at 90 °C and weighed. Working in a fume hood, 1.5 mL of 12 M H_2_SO_4_ was added, and the solution was left to digest the residue for 1 h at 30 °C in a water bath, vortexing every 10 min. The residue was then filtered with glass microfibre filters of known weight and washed first with hot water (15 mL) and then with acetone (15 mL). The residue plus filter was dried and weighed together. Then, it was ashed at 450 °C and weighed again (filter plus ash). The acid detergent lignin is the difference between the weight plus filter before and after ashing, with a correction for the weight loss of the filters themselves, which is determined separately.

### 2.7. Statistical Analyses

Experimental data were analyzed using SPSS 17.0 software (SPSS Inc., Chicago, IL, USA). The statistical significance of the difference in parameters related to plant growth, biomass accumulation and allocation, and gas exchange was evaluated by *t*-tests. The significant individual differences among the means of other parameters of different treatments were determined by Tukey’s multiple range tests after conducting homogeneity tests for variances. Differences were considered statistically significant at the *p* < 0.05 level. The Mantel test was used to examine the relationships between the major plant parameters and soil shear stress to assess the legacy effects of drought on plants and thereby on plant reinforcement to the soil.

## 3. Results

### 3.1. Legacy Effects of Drought on Root Reinforcement

**Figure 2:**
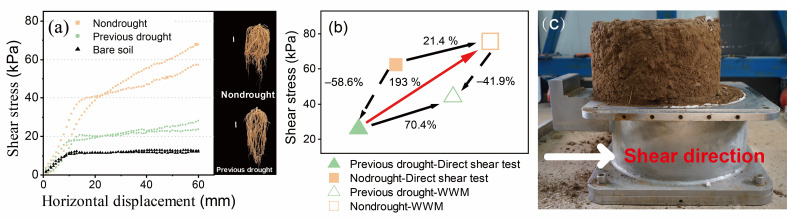
(**a**) Stress–strain curves obtained for bare and rooted soils. Vegetation significantly enhanced soil shear strength compared to bare soils, while drought-stressed plants develop deeper root systems, but with relatively lower root biomass. (**b**) Comparison of soil shear stress values measured by direct shear tests and those predicted by the Wu and Waldron Model (WWM). (**c**) Failure characteristics of the direct-shear root-permeated sample. Non-drought group: the soil was conditioned by *Amorpha fruticosa* plants assigned to an average rainfall treatment receiving 70 mm of water per month. Previous drought group: the soil was conditioned by *A. fruticosa* plants assigned to a drought treatment with no irrigation for 60 days followed by a rewetting treatment. ■ and ▲, respectively, represent the soil shear stress values of the non-drought group and previous drought group obtained from direct shear tests; □ and △, respectively, represent the soil shear stress values of the non-drought group and previous drought group predicted by the WWM.

Vegetation significantly enhanced soil shear strength compared to bare soils, regardless of prior drought exposure (Figure 2a). However, plants that had experienced a predrought treatment showed a marked decline in root reinforcement, as indicated by reduced soil shear stress. Direct shear tests revealed that predrought treatments led to a 58.6% reduction in soil shear stress, yielding a value of 25.8 kPa, contrasting with the stress observed in non-drought-exposed soils.

Using the WWM, notable differences in soil shear stress trends were observed between the two treatments (Figure 2b). Predicted shear stress of soils without predrought treatment showed a smaller 21.4% increase compared to measured values. Conversely, the predicted shear stress of root-permeated soil with predrought treatment is overestimated by 70.4%. Particularly, comparing the predicted value without predrought to the measured value with predrought reveals an overestimation of up to 193%.

### 3.2. Legacy Effects of Drought on Plant Morphological Traits and Gas Exchange

Although the growth of plants subjected to predrought treatment recovered following rehydration treatment, and parameters such as plant height, leaf mass, and root/shoot ratio resembled those of normal plants, their growth performance exhibited significant alterations.

As presented in Table 1, predrought treatment led to a significant reduction in plant DBH (34.3% reduction, *p* < 0.05) and biomass accumulation (33.9% reduction, *p* < 0.01). Notably, root biomass accumulation decreased by 40.1% (*p* < 0.01) due to predrought treatment, subsequently influencing root morphological characteristics.

**Figure 3:**
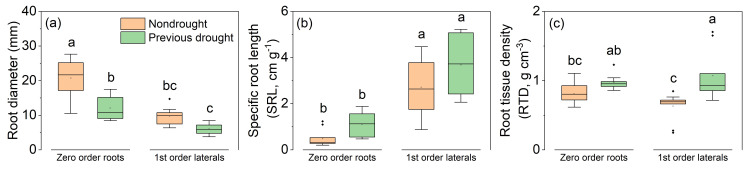
Legacy effects of drought on (**a**) root diameter, (**b**) specific root length, and (**c**) root tissue density in *A. fruticosa* plants. The bars with different letters are significantly different from each other (n = 10, *p* <0.05).

**Figure 4:**
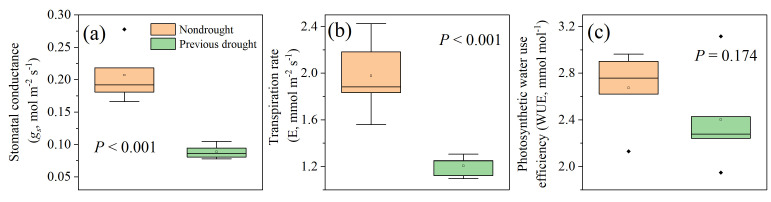
Legacy effects of drought on (**a**) stomatal conductance, (**b**) transpiration rate, and (**c**) photosynthetic water use efficiency in *A. fruticosa* plants.

Further analytical investigations were conducted to examine the drought legacy effect on root characteristics (Figure 3). After drought-rewetting treatment, the diameter of the first-order laterals in plants that had been pre-treated with drought returned to a normal level, exhibiting no significant difference when compared to plants that had not undergone previous drought treatment. However, a significant reduction (*p* < 0.05) was observed in the diameter of zero-order roots (Figure 3a). Concerning SRL, there was no significant variation detected post-rewetting treatment between zero-order or first-order laterals when contrasted with roots that had not experienced drought treatment (Figure 3b). In addition, after the drought-rewetting treatment, the RTD of first-order laterals in pre-treated plants significantly increased, whereas the RTD of zero-order roots showed negligible difference when compared to zero-order roots that had not been exposed to drought treatment (Figure 3c). Moreover, Figure 4 showed that plants subjected to predrought treatment exhibited significantly lower values for *g_s_* and *E*, while their photosynthetic WUE remained relatively unchanged when compared to normally grown plants.

### 3.3. Legacy Effects of Drought on Root Chemical Traits

Predrought treatments significantly affected the accumulation of root C and N. After drought-rewetting, the RCC of the first-order laterals in predrought-treated plants returned to normal (no significant difference from non-drought-treated plants), but the RCC of zero-order roots and the RNC in both zero-order and first-order laterals decreased significantly. This led to a significant increase in the root C/N ratio of both root types.

**Figure 5:**
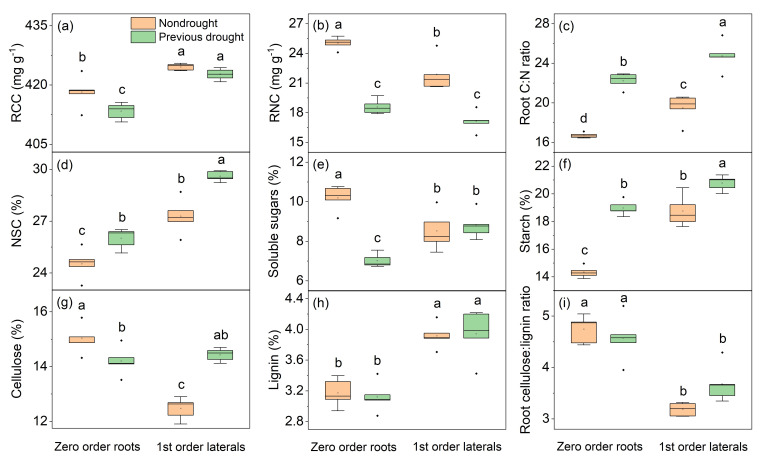
Legacy effects of drought on the parameters of root chemistry in *A. fruticosa* plants. (**a**) root carbon concentration (RCC); (**b**) root nitrogen concentration (RNC); (**c**) root C:N ratio; (**d**) nonstructural carbohydrate concentration (NSC); (**e**) soluble sugars concentration; (**f**) starch concentration; (**g**) cellulose concentration; (**h**) lignin concentration; and (**i**) root cellulose:lignin ratio. The bars with different letters are significantly different from each other (n = 5, *p* < 0.05).

The NSC content in plant roots increased notably after predrought treatment, with a similar trend in zero-order roots and first-order laterals. The NSC content in first-order laterals was higher than that in zero-order roots (*p* < 0.001, Figure 5d). Predrought treatment had a relatively small impact on the soluble sugar content (a key NSC component), with a decrease only in zero-order roots (*p* < 0.001, Figure 5e), but starch accumulation in roots was significantly affected. Both zero-order roots and first-order laterals showed a significant increase in starch content (Figure 5f).

Predrought treatment mainly changed the cellulose content rather than the lignin content or the cellulose/lignin ratio in the roots (Figure 5g–i). Specifically, the cellulose content of zero-order roots decreased significantly after drought-rewetting, while that of first-order laterals increased significantly (*p* < 0.001, Figure 5g).

**Figure 6:**
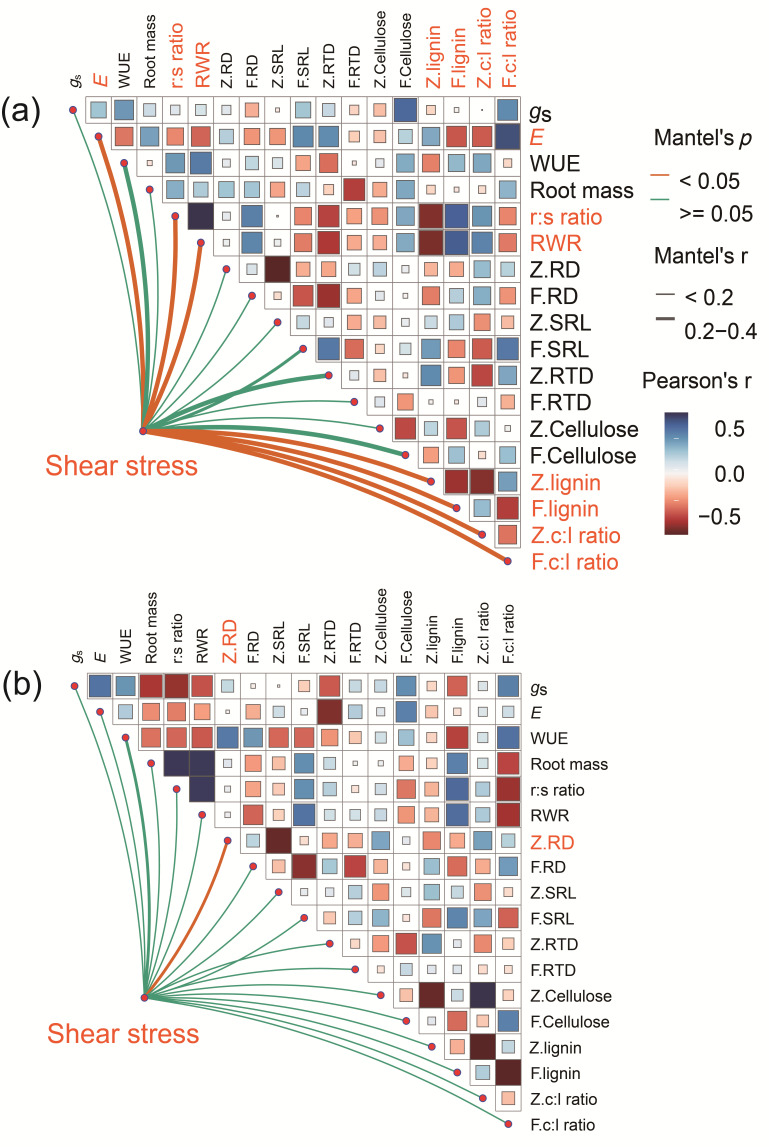
Mantel test correlations represent the relationship between plant traits and soil shear stress under (**a**) nondrought and (**b**) previous drought treatment conditions, respectively. The width and color of lines indicate Mantel’s *p*-value and Mantel’s *r*-value, respectively. Lines with Mantel’s *p* < 0.05 indicate significant correlations. *g*_s_, stomatal conductance; *E*, transpiration rate; WUE, water use efficiency; r:s ratio, root/shoot dry mass ratio; RWR, root weight ratio; Z (F). RD, the diameter of zero-order roots (first-order laterals); Z (F). SRL, specific root length of zero-order roots (first-order laterals); Z (F). RTD, root tissue density of zero-order roots (first-order laterals); Z (F). c:l ratio, cellulose/lignin ratio of zero-order roots (first-order laterals).

Mantel test analyses revealed significant variations in the correlations between plant traits and soil shear stress under different treatment conditions. When plants were not subjected to drought treatment, multiple plant traits, such as *E*, the root/shoot dry mass ratio, RWR, lignin, and the cellulose/lignin ratio, exhibited remarkable correlations with soil shear stress (Figure 6a). This indicates that these plant traits under normal conditions have a considerable impact on soil shear stress. However, after predrought treatment, the situation was notably different. It was found that only the diameter of zero-order roots had a strong correlation with soil shear stress (Mantel’s *p* = 0.014, *r* = 0.35; Figure 6b).

## 4. Discussion

Using data from China (2010–2012), Chen et al. [1] suggested a close relationship between debris flow events and previous drought. Soil mechanics researchers have emphasized that after a prolonged drought, rainfall onset causes a significant increase in pore-water pressure and a concomitant decrease in effective stress within the soil. These alterations lead to a weakened soil strength, making the soil more prone to collapse and debris flow. However, as an important element of soil reinforcement, changes in plant properties during the drought-rewetting process are often ignored [44], especially when evaluating the soil strength by various models. In this study, *A. fruticosa* plants were used as experimental materials to investigate the legacy effects of drought on soil shear strength and on mechanical and hydrological reinforcement-associated plant parameters. Our findings support the hypothesis that the adaptive strategies of plants during drought-rewetting process weaken their ability to maintain soil shear strength.

As shown in Figure 2a, the predrought treatment resulted in a substantial decrease in the shear stress of root-permeated soil. From the excavated root system, it was found that roots exhibited morphological changes, less biomass, and a tendency to grow into deeper soil compared to those without the predrought treatment. Fry et al. [16] suggested that plasticity and biomass allocation shifts in different ways according to root type, presumably to optimize limited resources. On the one hand, deeper roots with reduced branching angles can efficiently capture water from soil that is dry at the surface but retains moisture in deep layers [45]. On the other hand, deeper growing roots cross the potential soil shear surface and soil shear strength should be enhanced [6,28,46]. However, while *A. fruticosa* plants subjected to predrought treatment had deeper roots than plants not treated with previous drought, the shear strength of the root-permeated soil was lower. This suggested a complex of root reinforcement, and the use of a single trait to predict soil shear strength is not appropriate [10,47,48]. In addition, a significant decrease in soil shear strength can have far-reaching ecological consequences. For instance, the heightened susceptibility to landslides is a major concern [49,50]. With a weaker soil structure, even relatively mild seismic activities or heavy rainfall events could more easily trigger landslides. This poses a direct threat not only to the vegetation cover on the slopes but also to the surrounding habitats and ecosystems. In the present study, we showed the difference between the model predictions and the results of the direct shear tests. Briefly, the predictions made by the WWM overestimated the values by 21.4%, even to 193% when compared to those measured by direct shear tests (Figure 2b). Not only did this indicate that the predicted values of WWM overestimated the additional cohesion contributed by roots, but the magnitude of this overestimation was even more pronounced in plants that had undergone the drought-rewetting process. In other words, the legacy effects of drought could have a significant impact on the accuracy of root reinforcement prediction models developed based on plant traits. Previous studies have extensively discussed the pros and cons of various prediction models, including the WWM [34,51,52]. For example, the WWM generally considered that the distribution of roots in soil is uniform, but in reality, the distribution of roots in soil is very complicated. Moreover, it assumes that all roots fail simultaneously, which ignores the fact that when the soil is sheared, some roots slide out of the soil directly without breaking. In the following discussion, our focus is primarily on highlighting the changes in plant traits associated with soil reinforcement. These changes may provide new insights for optimizing models from the perspective of root plasticity. Root plasticity, the ability of plant roots to adapt morphologically, physiologically, and biochemically to environmental changes, may impact soil shear strength [49,50]. In relation to its contribution to reduced strength, key aspects include that drought-exposed plants’ plastic roots can alter the architecture, growing longer, thinner, and more branched. However, excessive branching may disrupt soil structure and weaken reinforcement, reducing overall shear strength. In addition, roots growing non-perpendicular to slopes due to plasticity are less effective in preventing soil slippage than those in ideal reinforcement directions. Indeed, roots can not only adjust to changes in the external moisture conditions within hours [53], but they have also evolutionarily acquired plasticity in their pursuit of water with respect to the geometric arrangement of individual roots and their three-dimensional organization within the soil [8,17]. For example, Fry et al. [16] compared the responses of root architectural classes of various plant species to the location of limited water in the soil column and indicated that root architecture governs plasticity in response to drought. Therefore, we suggested that more details about root properties, including variations in morphological, architectural, physiological, mechanical and biochemical properties induced by the adaptive strategies adopted by plants to water availability during the drought-rewetting process [44] should be considered. These changes may be more helpful in revealing the true cause of the reduced shear strength of the root-permeated soil than the root characteristics themselves.

In terms of appearance, *A. fruticosa* plants showed remarkable resilience during the drought-rewetting scenario, exhibiting similar plant height, leaf biomass, and root/shoot ratio as plants without predrought treatment (Table 1). However, the legacy effects of drought did indeed influence plant growth performance. In the present study, predrought treatment significantly reduced plant DBH. Cislaghi et al. [54] suggested that the root reinforcement of a tree was correlated with the DBH. Deljouei et al. [55] compared the differences in RAR as a function of DBH for *Carpinus betulus* and *Fagus orientalis*, revealing that the largest DBH class of both species had the highest RAR value. In other words, the greater the RAR, the more soil shear strength will be increased. Flepp et al. [56] demonstrated that there is a positive linear relationship between the mean DBH of spruce stands in Europe and the reinforcement of lateral roots. In addition, there is a strong allometric relationship between DBH and crown diameter in healthy trees in the young to mature stages of their growth [57]. Thus, the legacy effects of drought on *A. fruticosa* plants’ DBH might hamper root reinforcement and the canopy’s rainfall retention capacity.

The response of root biomass to drought shows great variability, which can potentially result in increased, decreased, or delayed responses. This variability is often linked to the intensity and duration of drought [58]. The predrought treatment caused a remarkable decrease in root biomass (Table 1). Additionally, according to Stokes et al. [59], roots of *A. fruticosa* plants were mainly defined as two classes, namely thick roots (>10.0 mm, zero-order roots) and thin roots (>2.0–10.0 mm, first-order lateral roots). In the current study, the diameter of both thick roots and thin roots consistently decreased (Figure 3a). Ma et al. [60] proposed that root diameter most influenced root trait variation across plant species, growth forms, and biomes. Root diameter distribution, together with RTD, is a trait that underlies variation in SRL, thereby affecting a plant′s ability to explore the soil [36]. The legacy effects of drought led to an increase in SRL of thick roots (Figure 3b), while an increase in RTD was found only in thin roots (Figure 3c). Higher SRL indicates that thick roots became thinner, while higher RTD suggests that thin roots experience a decrease in their tensile strength. Due to the different reinforcement roles of thick and thin roots, it was suggested that the legacy effects of drought on root reinforcement should be considered differently. Specifically, as root diameter is the most commonly used plant trait/parameter in root reinforcement and soil stabilization models, it is particularly important to summarize the characteristics of the plastic response of root diameter and the range of variation in changing environments.

In this study, *A. fruticosa* plants exposed to a predrought stress event showed only partial recovery following a subsequent rewetting treatment, indicating that the legacy effects of drought still limited several plant parameters in addition to growth, such as plant gas exchange. It was observed that these plants displayed lower *A*, *g_s_*, and *E* (Figure 4). This finding is consistent with the negative impact of drought stress on plant photosynthesis reported in previous studies [61,62,63]. Additionally, it has been proposed that recovery of gas exchange after natural drought is rapid unless limited by loss of leaf hydraulic conductance [64]. The process of root water uptake, closely linked to hydrological reinforcement by roots, relies on plant gas exchange, particularly transpiration. During moderate rainfall events, plants efficiently extract water from the deeper layers of the soil, leading to a decrease in soil moisture content. Consequently, soil suction intensifies and the soil’s capacity to retain water changes [65,66]. Prior to rainfall, evapotranspiration may further decrease soil moisture, allowing for greater water storage in the soil. This, in turn, delays the saturation levels at which landslides typically occur [7]. However, it should be noted that modifying the soil moisture regime through evapotranspiration might not have a significant impact on shallow landslides and debris flows that predominantly happen during prolonged wet seasons, where precipitation consistently surpasses potential evapotranspiration levels [59].

Apart from the physiological adaptations that plants undergo in response to drought stress, there are notable modifications in the chemical composition of the root system (Figure 5). These alterations include changes in nutrient content, cellulose, lignin, and other components. Some of the chemical changes are closely associated with the mechanical reinforcement by roots. For instance, N concentration displayed a consistent decrease trend in both thick roots and thin roots (Figure 5b), which may suggest that the capacity for root reinforcement decreases due to the restriction of root system development. In addition, there are many types of NSC compounds, and research has focused on carbohydrates, in particular soluble sugars and starch. O’Brien et al. [38] demonstrated a positive relationship between NSCs and drought survival. In our study, we observed an increase in NSCs in both thick and thin roots (Figure 5g). This suggested that the legacy effects of drought continue to affect plant performance, and plants maintain higher levels of NSCs to cope with the adverse stress [67]. The regulatory patterns of soluble sugar and starch accumulation vary between thick and thin roots (Figure 5e,f), potentially contributing to their distinct roles in cellulose biosynthesis, which is crucial for reinforcing soil on unstable slopes. The contents of cellulose, lignin, and the lignin/cellulose ratio were associated with root tensile strength, which plays an important role in soil reinforcement and slope stabilization [14]. In general, the higher the cellulose content, the higher the root tensile strength. Figure 5g showed that cellulose content followed opposite trends in thick and thin roots, decreasing in thick roots and increasing in thin roots. This again indicated that the legacy effects of drought have distinct effects on different types of roots, ultimately affecting overall root reinforcement. In addition, the Mantel test reveals differences in the potential relationships between plant traits and soil shear stress (Figure 6). Under normal conditions, a variety of plant characteristics collectively influence soil structure and its mechanical properties, which in turn affect soil shear stress. However, drought pretreatment significantly modifies this relationship. Specifically, only the diameter of zero-order roots shows a strong correlation with soil shear stress after drought stress, indicating that these thicker roots are more effective in anchoring the soil and preserving its structural stability [15]. It is crucial to acknowledge the limitations of this study. The analysis was confined to a subset of plant traits, and unmeasured characteristics could also impact soil shear stress. Furthermore, the study considered only drought as a stressor, whereas plants in natural environments face a multitude of stresses, including salinity and high temperatures. Future research should explore how plant traits interact with soil shear stress under these various stress conditions.

Our research sheds light on key strategies for managing drought-stricken soils and boosting vegetation’s role in slope stability. Here are the highlights: (1) Regular soil tests: essential for identifying soil areas weakened by past droughts, which helps target our efforts more effectively. (2) Plant selection: Opt for varieties that can withstand drought while still providing strong root support. Prioritize selecting plants with stable root systems that will not compromise soil interaction during short-term droughts. (3) Watering at critical times: strategic irrigation during key periods can prevent plants from taking extreme measures that could weaken soil structure, thus maintaining the soil’s integrity. (4) Root composition: Plant species that maintain a high lignin/cellulose ratio in roots during drought are ideal for slope planting. This composition is linked to stronger root tensile strength, which is vital for slope stability.

## 5. Conclusions

Our findings clearly illustrate the alterations in the interaction between plant traits and soil shear stress, both in the presence and absence of predrought treatment. These findings emphasize the intricate relationship between plant physiological and biochemical features and soil mechanical properties under varying environmental conditions.

The legacy effects of drought significantly decrease the soil shear strength of root-permeated soils, even though plants exhibit remarkable resilience during a drought-rewetting situation. Moreover, the legacy effects of drought can endure for an extended period and continuously influence the intrinsic functional traits of plants.

The adaptive strategies that plants employ to cope with the drought-rewetting process may undermine the soil reinforcement capability of their root systems. Considering the characteristics and patterns of plants’ plastic responses to environmental change can enhance the accuracy of predictions made by models related to root reinforcement and soil strength prediction that are developed based on plant traits.

## Figures and Tables

**Table 1:** Legacy effects of drought on plant growth, biomass accumulation and allocation in *A. fruticosa* plants. Data are mean ± SE (n = 3). Asterisks indicated a statistically significant effect tested with *t*-test: *, *p* < 0.05; **, *p* < 0.01; ns, non-significant.

	Height (m)	DBH (mm)	Plant Mass (g)	Root Mass (g)	Stem Mass (g)	Leaf Mass (g)	Shoot Mass (g)	Root/Shoot Ratio
Non-drought	1.99 ± 0.09	10.7 ± 0.93	343 ± 14.2	179 ± 6.18	133 ± 11.3	31.2 ± 3.92	164 ± 13.7	1.10 ± 0.10
Previous drought	1.69 ± 0.12	7.00 ± 0.81	227 ± 8.96	107 ± 6.84	95.4 ± 1.51	24.1 ± 1.80	119 ± 2.45	0.90 ± 0.04
*F*-value	0.491	0.146	1.15	0.017	4.52	1.46	3.97	3.96
*p*-value	ns	*	**	**	*	ns	*	ns

## Data Availability

Data is contained within the article.
